# Action, Application and Value of Oleum Bucalypti E Follis (Eucalyptol)

**Published:** 1897-04

**Authors:** Adolph Sandner

**Affiliations:** M. D.; M. R. C. S., Lond.; Chicago, Ill.


### ACTION, APPLICATION AND VALUE OF OLEUM EUCALYPTI E FOLIIS (EUCALYPTOL.)

The important position which the ol. eucalypti e foliis (it shall be termed eucalyptol in this treatise) has taken in the materia medica must make it desirable for the general practitioner to become intimately acquainted with it and to have some exact knowledge of its physiological action; and the fact that I have during several years studied its therapeutic application in medicine and surgery prompts me to submit my experience to the profession, and to add some extracts from the authentic literature on the subject.

1. Eucalyptol and Putrefactive Processes.—The first experimental study of the antiseptic properties of eucalyptol was carried out by Siegen and Mees, although Gimbert before them had noticed that the blood of rabbits, which had been injected with eucalyptol, did not putrefy, and that the cadavers of the so-treated animals mummified. Siegen states that he made an emulsion of 3 in 1000 gum arabic in water. He divided 1000 cc. of this emulsion into three equal parts. Into the first test-tube (a) he put the simple emulsion; into the second (b) he put the emulsion and 0.45 grammes of quinine hydrochlor., and into the third (c) he placed the emulsion and 0.45 grammes of eucalyptol. One cc. of muscles was put into each tube. The three tubes were then placed into a temperature of 70 degrees F., and lightly corked. On the fourth day a and b were found to smell intensely foul, while c did not smell. The eucalyptol preparation also contained less bacteria than the two others, and the movement of them was slower.

Schulz, who treated fresh fibrin with a 0.01 per cent. solution of eucalyptol and carbolic acid respectively, found that the eucalyptol preparation was still smelling after eucalyptol on the tenth day, and contained only a few slowly moving rods under the microscope on the eighteenth day, while the carbolic acid preparation was found to be smelling only faintly after carbolic acid, and was shown to contain colonies of bacteria on the tenth day.

I have found that eucalyptol prevents the development of bacteria on the usual culture media in a dilution of 1 in 666, while a dilution of carbolic acid of 1 in 200 is necessary to bring about the same effect. This statement refers to saprophytes only, but according to Omeltschenko (*Centralblatt fuer Bacteriologie*, IX., page 815, '92, translated in the *Bacteriological World and Modern Medicine*, May 1892, No. 7), a corresponding action is exercised upon tubercle bacilli and pyogenic cocci.

2. The Action of eucalyptol upon the Red and White Blood Corpuscles.—If a drop of frog's blood be exposed to the vapors of eucalyptol, the red corpuscles become covered with radiating striæ, which are probably due to the contraction of the stroma. The nucleus gets more distinct, and the contour of the cells changes from a round into a rhombic shape whenever space permits of this change.

When a drop of eucalyptol is put on a glass slide and allowed to come into contact with the corpuscle, the latter disappears, the nucleus resisting longest.

Very much more interesting is the action upon the leucocytes. If they be brought into contact with a 0.1 per cent. emulsion of eucalyptol, they at once lose all their power of retractability. The cells retract their projections, assume a round shape, the contour gets darker and the protoplasm becomes of a granular appearance. Considering this action of eucalytol upon leucocytes to prevent their amœboid movement in relation to inflammation, Mees has studied the mesentery of a curarized frog, and found that forty-eight hours after exposure the circulation was uninterrupted; there was neither any mural implantation of leucocytes to be seen, nor could any of them be detected outside the vessels. Binz corroborates this experiment.

3. Clinical Application of Eucalyptol in Surgery.—This action of eucalyptol to prevent the emigration of leucocytes, combined with its high antiseptic power and its non-toxic quality, makes the remedy of paramount importance in the treatment of wounds, and especially does it deserve great consideration in those cases where large suppurating cavities have to be treated or where septic infection has occurred. There are two principal ways of using the drug. The first is the use of a dressing consisting of gauze soaked in a 4 to 5 per cent. solution of eucalyptol; the second is the use of a 3 in 1000 solution (better emulsion in gum arabic), as an irrigation for suppurative and tuberculous cavities.*

Empyemas, suppurative and tuberculous joints, tuberculous abcesses, and the peritoneal cavity in cases where irrigation is necessary, may be advantageously washed out with this irrigation; and I have found out that the continuous antiseptic action of eucalyptol, acting as it does by an alterative process of oxydation and deoxydation, is the best means of correcting septic suppurative and tuberculous processes, besides being absolutely non-poisonous.

Before now different writers have pointed out the use of eucalyptol in irrigating suppurative cavities. So Schulz, in a discussion on its clinical value, says: "In a case of psoas abscess, resulting from tuberculosis vertebrarum corporis

*I prepare an alcoholic solution of eucalyptol (1 ounce to 5 ounces of absolute alcohol). To six ounces of this solution I add 15 to 20 ounces (three-quarters to one pint) of hot water, and prepare the gauze by soaking it in this. The vessel has to be closed tightly so as to prevent the evaporation of the eucalyptol. The irrigation is made by adding three drachms. (or more when an open wound is irrigated) of the alcoholic solution to one pint of water at the time of use.

(spondylitis), the abscess was incised, the cavity drained, and a Lister's dressing applied. In spite of this treatment, septic infection occurred on the third day, the temperature rose, and on the removal of the bandage the abundant secretion was found to be very foul smelling. The cavity was then washed out with a 0.3 per cent. eucalyptol emulsion and a wet eucalyptol dressing was applied, the result being that the disgusting smell disappeared very rapidly, the fever vanished in a few days, and the patient could be discharged as cured after a few weeks. In this case one has to consider that, mixed infection having taken place, the patient's life was saved by the treatment with eucalyptol."

For the dressing I claim:

1. That it is simple of application, and that its strength be changed by simply altering the way of applying it. I merely put the gauze, prepared as directed, on the wound, cover it up with a piece of oiled silk, and fasten the whole with a bandage. When a stronger action is desired I put on the gauze some cottonwool, and then cover up with oiled silk. This prevents the evaporation of the eucalyptol more effectively, and provides against the access of air.

2. That it is the strongest antiseptic that can be used without any corrosive action being exercised upon the tissues.

3. That it is absolutely non-poisonous.

4. That its slightly stimulating power promotes the growth of healthy granulations.

Schulz, Siegen, Mees, Binz, and lately I, have had very good results following this dressing, the only failure under similar application being reported by Bassini, who, in seven cases, found the septic processes to be not altered and the eucalyptol too irritating. Since his report appeared I got to know that he used the crude, unrefined oil, and this is to my mind a sufficient explanation.†

4. Resume of the use of Eucalyptol in Surgery:—1. Eucalyptol prevents the formation of pus and septic infection by its strong germicidal action. Further, by its action upon the leucocytes it stops their emigration from the vessels during an inflammatory process, and prevents them from being acted upon by any positively chemotactic acting substance.

2. Eucalyptol is slightly stimulating, when a pure preparation is applied in the manner indicated (as a dressing), and is therefore promoting the growth of healthy granulations. Unrefined preparations will be found to prove too irritant and will, in themselves, be a source of inflammation.

3. In the form of the irrigation, eucalyptol is a safe and certain antiseptic to use in any cavity where there is fear of some of the antiseptic being retained. Although eucalyptol

†For the experiment and clinical use detailed in this monograph I used the preparation of Sander & Sons.

has three times the strength of carbolic acid, as far as its germicidal action is concerned, it is absolutely non-poisonous; nine grammes in twenty-four hours, per orem, had not the slightest harmful effect.

4. Eucalyptol in its pure state is perfectly painless when applied to a wound or over an unbroken surface. It is desirable to apply it in this manner to wounds of a very septic nature, to burns and scalds, where the quick formation of healthy granulations is aimed at, and in bruises, sprains, etc., where an inflammatory process is to be controled. The dressing must not be too occlusive when it is used in this way.

5. Therefore the application of eucalyptol is especially advisable in those cases in which the application of carbolic acid or bichloride of mercury solutions must appear as dangerous; viz., in young individuals, in debilitated subjects and in cases where a large absorbing surface admits of the entrance of a large quantity of the antiseptic into the system.

ADOLPH SANDNER, M. D.
M. R. C. S., Lond.

Chicago, Ill.

